# Zn Vacancy Formation Energy and Diffusion Coefficient of CVT ZnO Crystals in the Sub-Surface Micron Region

**DOI:** 10.1038/s41598-018-31771-1

**Published:** 2018-09-07

**Authors:** Narendra S. Parmar, Lynn A. Boatner, Kelvin G. Lynn, Ji-Won Choi

**Affiliations:** 1 Center for Electronic Materials, Korea Institute of Science and Technology, Seoul, 02792 Republic of Korea; 2 Materials Science and Technology Division, Oak Ridge National Laboratory, Oak Ridge, TN 37831 USA; 3 Department of Physics and Astronomy, Washington State University, Pullman, WA 99164 USA; 4 Center for Materials Research, Washington State University, Pullman, WA 99164 USA; 5 Department of Nanomaterials Science and Engineering, Korea University of Science and Technology, Daejeon, 34113 Republic of Korea

## Abstract

By using positron annihilation spectroscopy methods, we have experimentally demonstrated the creation of isolated zinc vacancy concentrations >10^20^ cm^−3^ in chemical vapor transport (CVT)-grown ZnO bulk single crystals. X-ray diffraction ω-rocking curve (XRC) shows the good quality of ZnO single crystal with (110) orientation. The depth analysis of Auger electron spectroscopy indicates the atomic concentrations of Zn and O are almost stoichiometric and constant throughout the measurement. Boltzmann statistics are applied to calculate the zinc vacancy formation energies (*E*^*f*^) of ~1.3–1.52 eV in the sub-surface micron region. We have also applied Fick’s 2^nd^ law to calculate the zinc diffusion coefficient to be ~1.07 × 10^−14^ cm^2^/s at 1100 °C. The zinc vacancies began annealing out at 300 °C and, by heating in the air, were completely annealed out at 700 °C.

## Introduction

Zinc oxide has been under intensive and long-term investigation in both the academic and industrial communities because of its varied actual and potential applications in electronic and optoelectronic-based devices. Point-defect engineering in this material has become of significant importance since theoretical and experimental results suggest that a zinc vacancy acts as a shallow acceptor and would, therefore, be of importance in forming a *p-n* junction for blue/UV LEDs. Accordingly, achieving an understanding of the thermodynamics and kinetics of intrinsic point defects in ZnO is of both fundamental and technological significance. Zinc migration has previously been considered, in particular, with respect to the degradation of varistor devices^1^ whose function is believed to proceed through the migration of intrinsic defects - most likely zinc interstitials. Gaining an understanding of zinc defect diffusivities is also of importance in order to control the formation of unwanted and compensating defects that are likely to contribute to the well-known difficulties in synthesizing *p*-type zinc oxide^2,3^. Until recently, the most prevalent way of creating zinc vacancies has been either by electron or laser irradiation^4,5^, i.e., by non-equilibrium thermodynamic processes that lead to the simultaneous creation of compensating defects such as zinc interstitials, oxygen interstitials, and oxygen vacancies. The creation of such defects is relatively hard to avoid and control. Recently, Parmar *et al*.^6^, reported the formation of high concentrations of isolated zinc vacancies (>10^20^ cm^−3^) in thermodynamic equilibrium – findings that can prove to be of importance in obtaining *p*-type ZnO by controlling the formation of zinc vacancies^6^, as zinc vacancies are shallow acceptors in ZnO crystal.

Zinc vacancies created by electron irradiation^7–9^ and laser radiation^10^, have been the subject of a number of previously conducted studies designed to measure the self-diffusion of zinc in ZnO^11^. The prior experimental data, however, exhibit a considerable spread that renders their interpretation difficult. In view of this situation, a theoretical approach can provide valuable insights into the various atomistic migration processes and, thereby, can help to quantify their respective contributions.

In this letter, we present results on the formation energy and diffusivity of zinc vacancies in ZnO, where a large number of zinc vacancies (10^17^ < V_Zn_ ~ 10^20^) are created in thermodynamic equilibrium by oxygen annealing. Having such a concentration of zinc vacancies in thermodynamic equilibrium provides the basis for carrying out a study of the formation energy and diffusion coefficient that is more reliable relative to an approach where zinc vacancies are created by a non-thermodynamic-equilibrium process such as, an electron irradiation or bombardment using a laser exposure.

## Experimental Methods

Chemical vapor-transport-grown ZnO crystals were placed in Heraeus high-purity quartz ampoules that were evacuated using roughing and turbo molecular pumps and that were then baked at ~150 °C overnight to remove residual water vapor. The vacuum pressure was ~10^−7^ Torr prior to back filling with ultra-high-purity oxygen to ~300 Torr. The ampoules were then sealed using an oxy-hydrogen torch and placed in a tube furnace. The annealing process was carried out at 1100 °C or 1200 °C for 24 hours, and the ampoule remained in the furnace during cooling.

Quenching experiments were also carried out by dipping the hot ampoule (1200 °C) in water at room temperature (RT). This quenching process reduced the ZnO temperature from 1200 °C to RT in a few seconds. Annealing (i.e., annihilation) out of the zinc vacancies was performed by heating the crystals in air for one hour at either 300 °C, 500 °C or 700 °C.

Positron annihilation spectroscopy (PAS)^12^ is a well-known tool to characterize negatively charged defects, such as zinc vacancies (*V*_*Zn*_) in ZnO crystals^13^. Positrons are positively charged and become trapped in negatively charged native defects, which reduces their Doppler momentum. The trapped positrons eventually annihilate the surrounding electrons, emitting two photons of 511 keV energy^14^. Emitted photons with Doppler broadening are the signature of the annihilation site. Though, PAS is not very useful for investigating positively charged defects, it has been quite helpful in investigating neutral defects^15^. The signal-to-noise ratio can be increased by a significant amount by performing two-detector coincidence measurements^16^. However, the coincidence-mode process takes a much longer time to collect data. In this letter, depth-resolved positron annihilation spectroscopy (PAS) Doppler broadening measurements were performed at the Washington State University (WSU). The 511 keV annihilation peak was recorded using a liquid-nitrogen-cooled HPGe detector. The *S* parameter is sensitive to the annihilation fraction with low-momentum valence electrons and is proportional to the concentration of trapping centers^17^. The *W* parameter comprises the wings of the peak where higher momentum Doppler shifts dominate, and it relates chemical species to the annihilation site. Together, the *S* and *W* parameters were used to characterize positron-trapping centers in the ZnO crystals. Further positron experimental details and analysis are discussed elsewhere^6,18^.

X-ray diffraction (XRD) measurement was carried out using a high-resolution ATX-G, Rigaku, triple-axis diffractometer system, using Cu Kα radiation, with a scintillation counter (0-D detector).

The elemental composition along the depth direction was measured by Auger electron spectroscopy (AES). The crystal surfaces were measured with an AES, PHI 700 (ULVAC-PHI, INC) system, and the accelerating voltage of the first exciting electron beam was 5 kV.

## Data Analysis

### X-ray diffraction (XRD)

**Figure 1:**
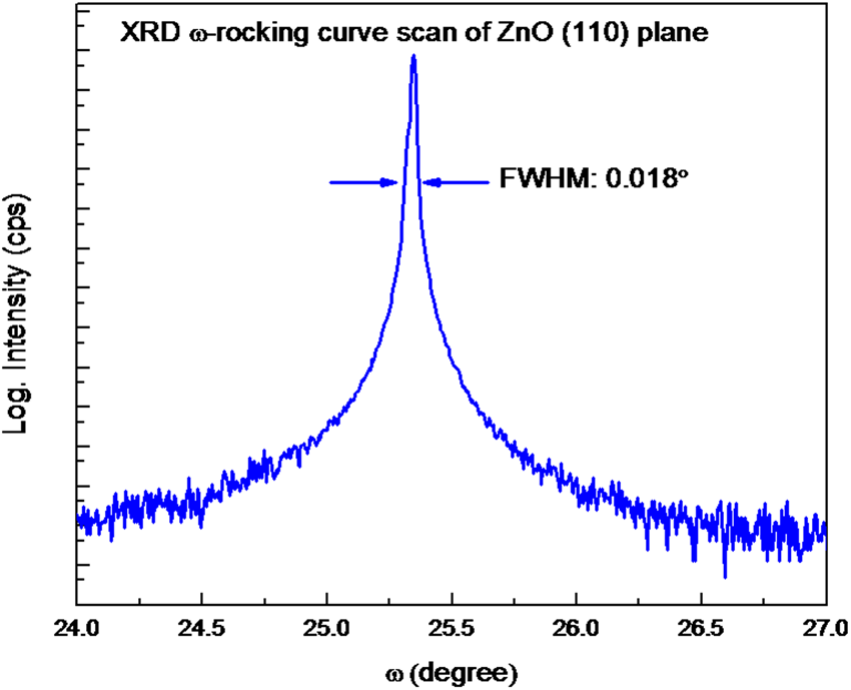
X-ray rocking curve (XRC) of the ZnO single crystal.

Figure 1 shows the X-ray diffraction ω-rocking curve (XRC) of the as-grown CVT ZnO single crystal. An omega scan was performed for the reflection from the (110) crystal surface. The full-width at half-maximum (FWHM) was measured to be 0.018°, indicating the good crystallinity of the CVT-grown ZnO single crystal. Electron back scattering diffraction (EBSD) measurement, also suggested a good quality of ZnO crystal, with a (110) plane orientation (not shown).

### Auger electron spectroscopy (AES)

**Figure 2:**
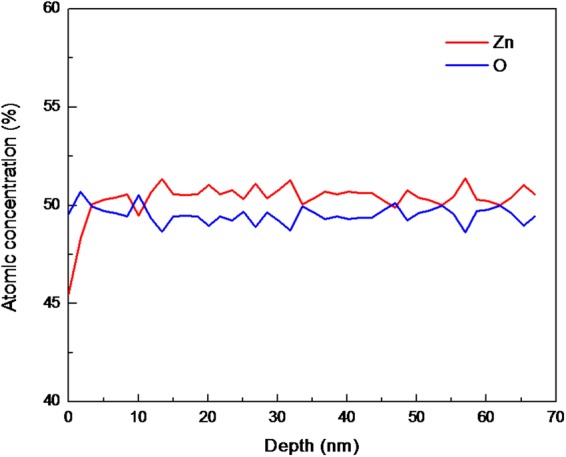
Auger electron spectroscopy (AES) depth profile spectra of an as-grown CVT ZnO crystal.

AES depth analysis was done by etching a ZnO crystal with an Ar-ion gun, and the variation of O(KLL) and Zn (LMM) intensity along the depth direction of the crystals was measured. In all the depth profiles, the main elements present in ZnO, i.e. zinc (Zn) and oxygen (O) were detected. As shown in Fig. 2 the atomic concentrations of Zn and O were found to be almost stoichiometric (Zn_0.505_O_0.495_) and constant throughout the measurement, which clearly indicates a chemically uniform and stoichiometric ZnO crystal.

### Defect concentration

The concentration of a point defect depends on its formation energy. In thermodynamic equilibrium and in the dilute regime (i.e., neglecting defect-defect interactions), the concentration of a point defect is given by

1 $${\rm{c}}={{\rm{N}}}_{{\rm{sites}}}\exp (-\frac{{{\rm{E}}}^{{\rm{f}}}}{{{\rm{k}}}_{{\rm{B}}}{\rm{T}}})$$

where, E^f^ is the formation energy, N_sites_ is the number of sites the defect can be incorporated on, k_B_ the Boltzmann constant, and T is the temperature. Equation (1) shows that defects with high formation energies will occur in low concentrations.

**Figure 3:**
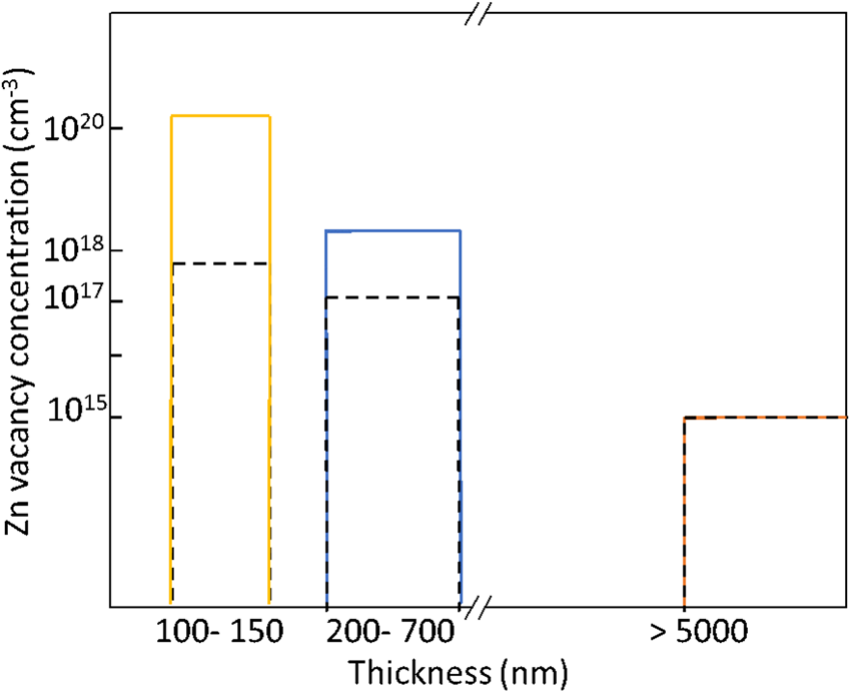
Schematic for the zinc vacancy concentration profile after oxygen annealing the CVT crystal at 1100 °C (broken lines) and 1200 °C (solid lines) (not to scale)^6^.

Annealing CVT-grown ZnO crystals at 1200 °C created ~5 × 10^20^ cm^−3^ defects in the top (100–150 nm) and ~1 × 10^18^ cm^−3^ in the mid (200–700 nm) crystal region. The bulk crystal region (>3 µm) remains with the minimal zinc vacancy concentration (~10^15^ cm^−3^),thereby matching the pristine state (i.e., as-grown) of the ZnO crystals^6^ [Fig. 3]. Details of the calculation of the zinc vacancy (*V*_*Zn*_) concentration are described elsewhere^6^.

### Zinc vacancy formation energy

The zinc vacancy formation energies were calculated using the Boltzmann Equation – Eq. 1. A CVT sample annealed at 1200 °C yields ${E}^{f}$ ~ 0.64 eV in the top region and ~1.39 in the mid region. The discrepency in these formation energies can be understood since Eq. 1 is only valid in the dilute limit. Positron measurements showed that, in the top region, the SW data deviated from a straight line - suggesting a saturation of positrons with excess (*V*_*zn*_) > 10^20^ cm^−3^ for the 1200 °C oxygen-annealed sample. The zinc vacancy formation energy (*E*^*f*^) is 1.38 eV (top) and 1.52 eV (mid) region for the 1100 °C annealed CVT sample. The deviation of ~0.14 eV for this sample in two regions is quite reasonable, and Eq. 1 can be assumed to be valid for the calculation of zinc vacancy formation energies, since (*V*_*zn*_) is <10^18^ cm^−3^. The zinc vacancy formation energies are summarized in the Table 1.

**Table 1:** Formation energy, where subscripts *a* and *b* denote the top and the mid regions, respectively.

T (K)	C_*a*_ (cm^−3^) (100–150 nm, top region)	C_*b*_ (cm^−3^) (200–700 nm, mid region)	${{\boldsymbol{E}}}_{{\boldsymbol{a}}}^{{\boldsymbol{f}}}$ (eV)	${{\boldsymbol{E}}}_{{\boldsymbol{b}}}^{{\boldsymbol{f}}}$ (eV)
1473	5.3 × 10^20^	1.5 × 10^18^	0.64	1.39
1373	7.2 × 10^17^	2.1 × 10^17^	1.38	1.52

### Diffusion coefficients

**Figure 4:**
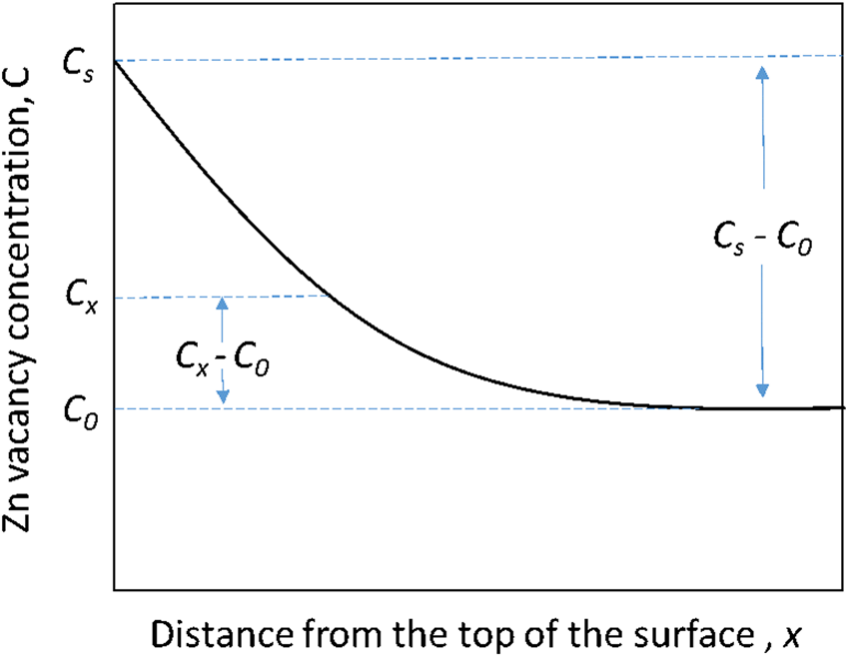
Demonstration of the Gaussian error function for the estimation of the diffusion coefficient.

The zinc diffusion coefficient at 1100 °C was calculated by using Fick’s 2^nd^ law:

2 $$\frac{{C}_{x}-{C}_{0}}{{C}_{s}-{C}_{0}}=1-{\rm{erf}}(z),\,{\rm{where}}\,{\rm{z}}=\frac{x}{2\sqrt{Dt}}$$

where, C is the zinc vacancy concentration [Fig. 4], erf(*z*) is the Gaussian error function, x is the diffusion distance, z is the approximated value of the Gaussian error function, D is the diffusion coefficient or diffusivity, and t is the diffusion time. The diffusion coefficients are summarized in the Table 2.

### Quenching

**Figure 5:**
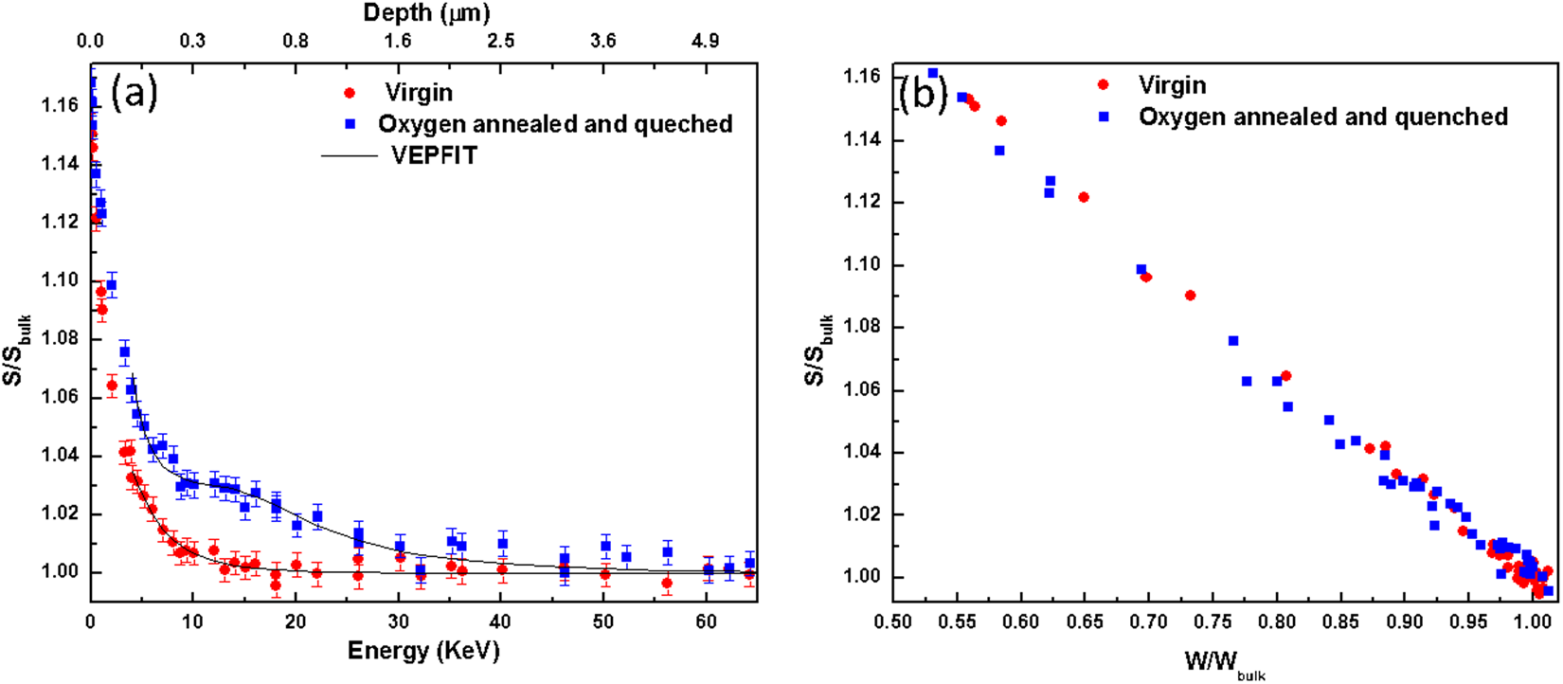
(**a**) Normalized S-vs-energy/depth (**b**) normalized S-W plot, in an as-grown (virgin) and oxygen annealed then quenched CVT ZnO crystal.

Positron depth-resolved data were fit (from 4 keV onwards) using the VEPFIT^19^ computer code. The S-parameter is ~3% higher with respect to the bulk in the top layer [Fig. 5(a)]. The positron diffusion length is 20 nm in the top layer. The S-W data [Fig. 5(b)] lie on a straight line suggesting the presence of one type of defect related to (*V*_*zn*_).

The zinc vacancy concentration (*V*_*zn*_) (atom^−1^) was calculated by using the equation:

$$c=\frac{{\lambda }_{b}}{\mu }\cdot \frac{{S}_{ave}-{S}_{b}}{{S}_{defect}-{S}_{ave}}$$

where, μ is the specific trapping rate, and [${\lambda }_{b}^{-1}={\tau }_{b}$] is the positron lifetime in the bulk semiconductor. The subscripts refer to the measured τ or S in the case of average (*ave*), the bulk value for (*b*) and the defect specific value in the case of *defect*. The ZnO atomic density was assumed *(ρ* ~ 8.3 × 10^22^ cm^−3^).

In a quenched sample, *S*_*ave*_ increased by only ~3%, i.e., a much lower value than for the slowly cooled samples - leading to a value of only (*V*_*zn*_) ~ 1.15 × 10^17^ cm^−3^ [Table 3]. Generally, it is believed that the actual concentration of vacancies will be higher than the equilibrium value if the crystal is annealed at an elevated temperature and then cooled suddenly, thereby freezing in the vacancies. Our observation, however, was exactly the opposite since the zinc vacancy formation energy in the quenched condition [Table 4] is somewhat higher than that compared to the slow cooling case. A precise comparison is not justified, however, since the quenching process does not follow thermodynamic equilibrium conditions.

**Table 2:** Diffusion coefficient calculation for CVT samples that were oxygen annealed at 1100 °C for 24 hours.

T (K)	C_*x*_ (200–700 nm, mid region)	C_*s*_ (100–150 nm, top region)	C_*o*_ (bulk)	$\frac{{{\bf{C}}}_{{\bf{x}}}{\boldsymbol{-}}{{\bf{C}}}_{{\bf{0}}}}{{{\bf{C}}}_{{\bf{s}}}{\boldsymbol{-}}{{\bf{C}}}_{{\bf{0}}}}$	erf (z)	z	x (cm)	D (cm^2^/s)
1373	2.5 × 10^−4^	8.5 × 10^−4^	1.2 × 10^−6^	2.93 × 10^−1^	0.70	0.74	4.5 × 10^−5^	1.07 × 10^−14^

Diffusion distance, *x* = 4.5 × 10^−5^ cm.

Where, C_*x*_, C_*s*_ and C_*o*_ values were calculated using the percentage ratio of the zinc vacancy concentration in their respective region to the atomic density (8.3 × 10^22^ cm^−3^). From Fick’s 2^nd^ law, the zinc diffusion coefficient at 1100 °C was calculated to be ~1.07 × 10^−14^ cm^2^/s.

**Table 3:** Zinc vacancy concentration in the quenched sample.

${{\boldsymbol{\lambda }}}_{{\boldsymbol{b}}}^{-{\bf{1}}}$(ps)	*μ* (s^−1^)	S_b_	S_defect_	S_ave_	c (atom^−1^)	C (cm^−3^)
170	3 × 10^15^	1	1.07	1.03	1.39 × 10^15^	1.15 × 10^17^

**Table 4:** Zinc vacancy formation energy under quenched conditions.

T (K)	C (cm^−3^) (500 nm–1 µm)	*E* ^*f*^ (eV)
1473	1.15 × 10^17^	1.71

### Annealing out zinc vacancies

**Figure 6:**
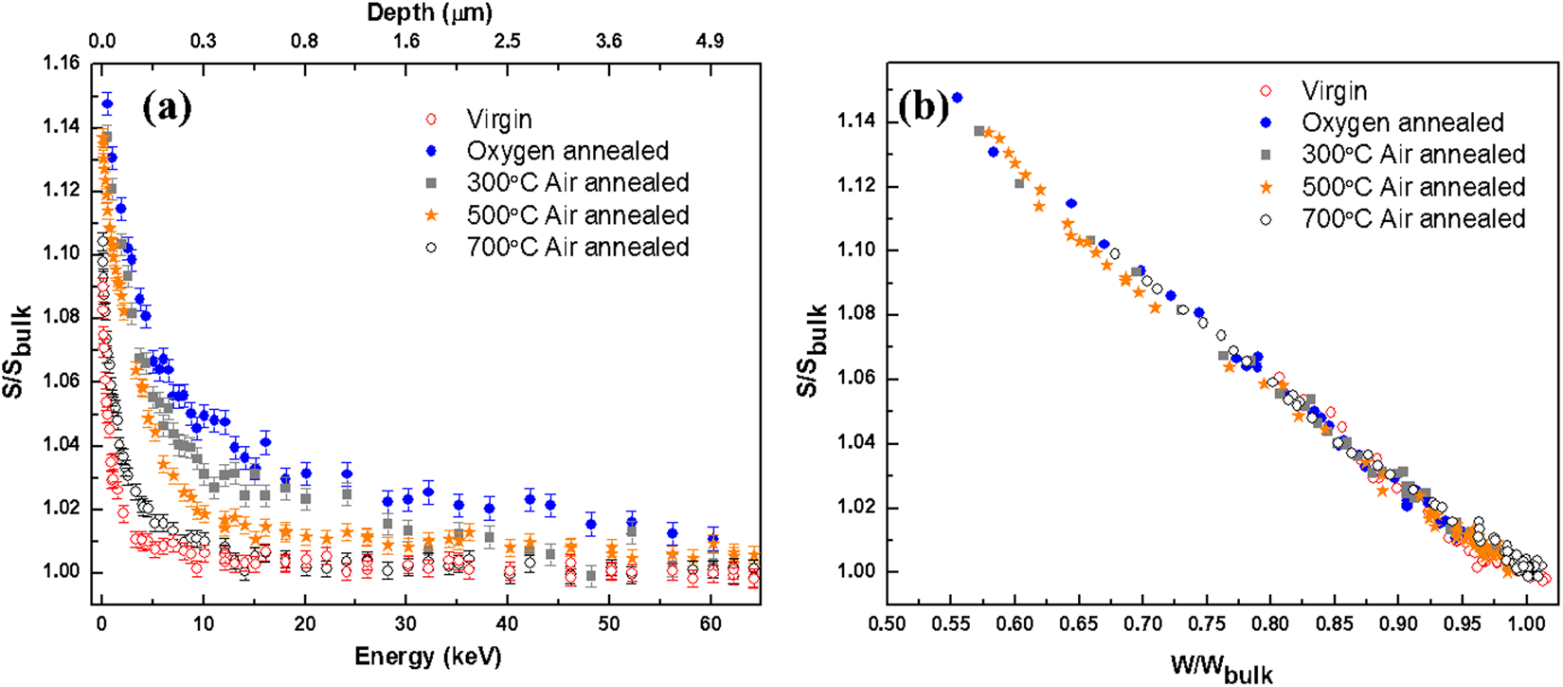
Positron data (**a**) (S vs Energy/depth) (**b**) S vs W, of an air-annealed (1 hour) CVT crystal that was then oxygen annealed at 1100 °C.

A CVT crystal, oxygen annealed at 1100 °C, [(*V*_*zn*_) ~ 1 × 10^18^ cm^−3^ (top layer)], was air annealed at various temperature steps (300 °C, 500 °C and 700 °C) for 1 hour each and this procedure was followed by performing the positron measurements [Fig. 6] after every air-annealing step. The positron data suggest that the (*V*_*zn*_) completely anneals out (i.e., to the as-grown (virgin) level) after a 700 °C air anneal for 1 hour. The S-W data followed a straight–line trend suggesting that no zinc vacancy-related clusters were formed during the annihilation process.

### A comparative study

Janoti *et al*.^20^, have reported a zinc vacancy formation energy of ~3.7 eV using first-principles calculations under *p*-type conditions (*E*_*F*_ at the VBM), in an oxygen-rich atmosphere. Kohan *et al*.^21^, have suggested that the zinc vacancy formation energy is ~(Zn(0/−1) = 5.8 eV & Zn(−1/−2) = 6.6 eV) under a high zinc partial pressure and ~(Zn(0/−1) = 2.42 eV and Zn(−1/−2) = −0.2 eV) under a low zinc pressure condition - assuming *E*_*F*_ at the VBM. Various other first-principles calculations are summarized in the Tables 5 and 6.

**Table 5:** Calculated formation enthalpies for *V*_*Zn*_ defects in bulk zinc oxide for zinc-rich and *p*-type conducting conditions [E_F_ = 0 eV, VBM].

Defect	Charge state	Formation energy (eV)				
		ref.^21^	ref.^24^	ref.^25^	ref.^26^	ref.^27^
*V*_*Zn*_	−2	6.6	7.06	5.8	5.1	5.9
	−1	5.8	5.96	5.7	5.0	5.8
	0	6.0	5.6	5.8	≥5.1	6.0

ref.^21^; DFT, LDA, ultra-soft pseudopotentials; ref.^24^: GGA + U; ref.^25^: DFT, LDA, norm-conserving pseudopotentials; ref.^26^: DFT, GGA, ultra-soft pseudopotentials; and ref.^27^: DFT, LDA, norm-conserving pseudopotentials.

**Table 6:** Calculated formation enthalpies for *V*_*O*_ and *Zn*_*i*,*oct*_ defects in bulk zinc oxide for zinc-rich and *p*-type conducting conditions [E_F_ = 0 eV, VBM].

Defect	Charge state	Formation energy (eV)				
		ref.^21^	ref.^24^	ref.^25^	ref.^26^	ref.^27^
*V*_*O*_	0	0	1.71	1.5	—	0.9
	+1	0.2	0.71	0.8	—	—
	+2	−0.3	−0.73	−0.5	−0.9	−0.5
*Zn*_*i*, *oct*_	0	1.7	4.25	3.4	1.2	—
	+1	1.3	1.69	1.5	≥0.4	—
	+2	0.9	0.02	−0.2	−0.6	—

Also, Lany *et al*.^22^, have performed first principle calculations and showed that the intrinsic defects (V_*O*_ and Zn_*i*_) can’t lead to shallow donors due to a large formation energy, when E_*F*_ is close to the CBM. Tomlins *et al*.^11^, performed zinc self-diffusion experiment in single crystal ZnO using nonradioactive ^70^Zn as the tracer isotope and reported zinc vacancies formation energy >3.51 eV. Azarov *et al*.^23^, did zinc-diffusion measurements using isotopically modulated ZnO crystals and showed zinc vacancies formation energy varies as, ${E}_{VZn}^{f}=1.1-2\times ({E}_{F}-{E}_{C})$, (where, (*E*_*F*_ − *E*_*C*_) is the difference in the Fermi level and conduction band energy) and depends on the position of the Fermi level. It can be seen that there exist a large discrepancies in zinc vacancies formation energy values calculated theoretically or experimentally.

Based on the positron analysis for the zinc vacancy concentration, the ZnO crystal region was divided into 3 parts: (1) top region (100–150 nm), (2) mid-region (200–700 nm) and, (3) bulk region (>3 µm). Our calculated values are based on the near-surface (~100–700 nm) experimental data (*V*_*zn*_), that requires less formation energy as compared to the bulk region. The calculated (*V*_*zn*_) formation energy is much lower than any previously reported values. This was observed experimentally, since there was no increase in the *V*_*zn*_ concentration in the bulk region, which is consistent with the high formation energy *V*_*zn*_ in the bulk region.

## Conclusions

The good quality of ZnO crystal with (110) orientation was used in this study as suggested by X-ray diffraction ω-rocking curve (XRC). The depth analysis of AES indicates the atomic concentrations of Zn and O are almost stoichiometric and uniform throughout the measurement. In general, the diffusivity is greater through the less restrictive structural regions, such as the near surface (micron region), as compared to the bulk. Based on the zinc vacancy concentration and using Fick’s 2^nd^ law, we have calculated zinc diffusion coefficient of ~1.07 × 10^−14^ cm^2^/s at 1100 °C in the sub-micron region. The zinc vacancy formation energy (*E*^*f*^) is calculated to be 1.38 eV (100–150 nm) and 1.52 eV (200–700 nm) region for 1100 °C oxygen anneal samples. These values are significantly lower than the reported values as obtained by first-principles calculations based on the bulk region of the ZnO crystal and few other reported experimental results.
